# Associations between Gut Microbiota and Intestinal Inflammation, Permeability and Damage in Young Malawian Children

**DOI:** 10.1093/tropej/fmac012

**Published:** 2022-02-12

**Authors:** Emma Kortekangas, Yue-Mei Fan, David Chaima, Kirsi-Maarit Lehto, Chikondi Malamba-Banda, Andrew Matchado, Chilungamo Chingwanda, Zhifei Liu, Ulla Ashorn, Yin Bun Cheung, Kathryn G Dewey, Kenneth Maleta, Per Ashorn

**Affiliations:** 1 Center for Child, Adolescent and Maternal Health Research, Faculty of Medicine and Health Technology, Tampere University, Tampere 33014, Finland; 2 School of Public Health and Family Medicine, College of Medicine, University of Malawi, Blantyre, Malawi; 3 Department of Nutrition and Institute for Global Nutrition, University of California Davis, Davis, CA 95616, USA; 4 Program in Health Services & Systems Research and Centre for Quantitative Medicine, Duke-NUS Medical School, Singapore 169857, Singapore; 5 Department of Pediatrics, Tampere University Hospital, Tampere 33520, Finland

**Keywords:** gastrointestinal microbiome, environmental enteric dysfunction, child health, leukocyte L1 antigen complex, REG1B, alpha 1-antitrypsin

## Abstract

**Background:**

Environmental enteric dysfunction (EED) is common in low- and middle-income countries and associated with childhood undernutrition. The composition of gut microbiota has been implicated in the pathogenesis of EED. Our aim was to assess the associations between gut microbiota and EED biomarkers in rural Malawian children. We hypothesized that there would be an inverse association between microbiota maturity and diversity and fecal concentrations of EED biomarkers.

**Methods:**

We used data from fecal samples collected at 6, 18 and 30 months from 611 children who were followed up during a nutrition intervention trial. The primary time point for analysis was 18 months. Microbiota data were obtained through 16S rRNA sequencing and variables included microbiota maturity and diversity, phylogenetic dissimilarity and relative abundances of individual taxa. EED biomarkers included calprotectin (marker of inflammation), alpha-1 antitrypsin (intestinal permeability) and REG1B (intestinal damage).

**Results:**

There was an inverse association between microbiota maturity and diversity and fecal concentrations of all 3 EED biomarkers at 18 months (*p*≤0.001). The results were similar at 30 months, while at 6 months inverse associations were found only with calprotectin and alpha-1 antitrypsin concentrations. At 18 months, EED biomarkers were not associated with phylogenetic dissimilarity, but at 6 and 30 months several associations were observed. Individual taxa predicting EED biomarker concentrations at 18 months included several Bifidobacterium and Enterobacteriaceae taxa as well as potentially displaced oral taxa.

**Conclusions:**

Our findings support the hypothesis of an inverse association between microbiota maturity and diversity and EED in rural Malawian children.

## INTRODUCTION

Several studies have focused on environmental enteric dysfunction (EED) as a potential underlying factor of childhood undernutrition [1–5]. In EED, a high pathogen load caused by fecal–oral contamination is thought to lead to intestinal damage and permeability, bacterial translocation and intestinal and systemic inflammation, ultimately contributing to impaired growth and development [6–10]. The exact mechanisms are unclear, and more insights into the determinants and consequences of EED are needed to improve child health outcomes in low-income settings, in which children are at a high risk for undernutrition [11].

Recent studies suggest that intestinal microbiota composition, which has been linked with undernutrition, may be associated with EED [12, 13]. Based on experimental studies in rodents, ingested pathogens could directly cause EED and a relative reduction in anti-inflammatory intestinal bacteria could aggravate the condition [14, 15]. On the other hand, EED could alter the microbiota composition, for example through immune activation [16]. However, few studies have investigated the associations between EED and microbiota in humans [15, 17].

The aim of the present study was to investigate how the gut microbiota is associated with markers of intestinal inflammation, permeability and damage (EED biomarkers) in rural Malawian children at 6, 18 and 30 months of age. We used data from 611 children who were followed up during and after a nutrition intervention trial (iLiNS-DYAD Malawi, NCT01239693) [18, 19]. We hypothesized that there would be an inverse association between microbiota diversity and maturity and EED biomarkers [13, 20]. Additionally, we conducted secondary exploratory analyses on associations of beta diversity and taxon-level microbiota composition with those biomarkers.

## MATERIALS AND METHODS

### Study sample

The data for this study were collected during the iLiNS-DYAD trial, which was a randomized-controlled nutrition intervention trial conducted in 2011 to 2015 in a mostly rural area in the Mangochi district in southern Malawi. The main outcome of the trial was length-for-age Z-score (LAZ) at 18 months on which no positive effect of the intervention was found [18, 19]. There was also no consistent effect of LNS on the gut microbiota or EED, but LNS was associated with higher microbiota diversity at 18 months.(Z. Liu, submitted for publication and [21]) The trial enrolled 1391 pregnant women who were randomized to 1 of 2 intervention groups or a control group. The intervention groups received either a small-quantity lipid-based nutrient supplement (LNS) or multiple-micronutrient tablets (MMN) until 6 months after delivery while the control group received iron and folic acid during pregnancy and placebo for 6 months after delivery. The first 869 enrolled mother–child dyads were assigned to a complete follow-up scheme (288 in the LNS, 291 in the MMN and 290 in the control group), in which they were monitored closely during pregnancy and the first 18 months after birth and children in the LNS intervention group received LNS from 6 to 18 months. Most of these participants were additionally followed up until 30 months. The intended sample size of 864 was calculated based on the assumed effect size of the main outcome of the trial and an estimated loss to follow-up of up to 25% [19]. This sample size gave 72% power to detect a partial correlation of 0.1 for associations between microbiota maturity and diversity and EED biomarkers with a 2-sided type 1 error rate of 0.05.

### Sample collection and processing

Data from fecal samples collected as described previously at 6, 18 and 30 months from children in the complete follow-up group were used for the current study [22]. Microbiota data were obtained through 16S sequencing of the frozen fecal samples using previously described methods [13, 23, 24]. Briefly, the samples were cryo-pulverized using liquid nitrogen, suspended and shaken in a bead beater (BioSpec Products, Bartlesville, OK) to mechanically disrupt bacterial cells. DNA was purified by centrifuging, precipitation and binding to a silica membrane that was washed with elution buffer (QIAquick column, Qiagen, Germantown, MD). The amount of DNA in all samples was normalized and the 16S V4 region of the bacterial DNA was amplified by PCR using primers with a barcode sequence unique to each sample [25]. After a second normalization step and pooling and purification of all samples, the amplicons were sequenced using an Illumina MiSeq instrument (version 2 chemistry, Illumina, San Diego, CA). Paired-end 250 bp reads were trimmed to 200 bp, combined and clustered according to 97% base pair identity using QIIME [26]. These clusters were each defined as an operational taxonomic unit (OTU) and sequences were aligned with PyNAST. The Ribosomal Database Project version 2.4 classifier was trained with a custom dataset of bacterial taxonomy and OTUs were assigned taxonomy mostly to genus or species level resolution [23, 27]. The relative abundance of each OTU in each sample was quantified by the number of sequence reads assigned to it. To exclude artefacts, OTUs were filtered with a threshold of 0.1% of sequencing reads in at least 2 samples. The V4-16S sequence data generated and analyzed for this study are available through the European Nucleotide Archive under the study accession number PRJEB29433.

For the measurement of EED biomarker concentrations, fecal samples were homogenized in extraction buffer and diluted. Calprotectin, alpha-1-antitrypsin and REG1B concentrations were measured from the sample supernatants using quantitative enzyme-linked immunosorbent assays (DH002 Fecal Calprotectin Assay, Hycult Biotech, Uden, The Netherlands; Human alpha1-Antitrypsin ELISA Kit, PromoCell GmbH, Heidelberg, Germany; and REG1B ELISA kit, TECHLAB, Inc., Blacksburg, VA, USA). For quality control, the first 100 measurements of calprotectin and alpha-1-antitrypsin were done in duplicate. All measurements of REG1B were done in duplicate and the mean of the 2 values was taken. If a value differed from the mean by more than 15%, the measurement was repeated.

### Variables

Microbiota variables included measures of microbiota maturity and diversity for primary analyses and beta diversity and relative abundances of bacterial taxa for secondary analyses. As a measure of microbiota maturity, the microbiota ages of the participants were estimated using a previously described Random Forests model [13, 24]. These microbiota ages were compared to the median of microbiota ages of an age-matched healthy reference cohort of Malawian children to obtain microbiota-for-age Z-scores (MAZ) [24]. For microbiota diversity, Shannon’s diversity Index was calculated from rarefied OTU-counts (rarefied to 5000 reads) using the R package phyloseq [28]. In addition, weighted and unweighted UniFrac distances were calculated to assess phylogenetic dissimilarity (beta diversity) between samples. Relative abundances of individual bacterial taxa were measured as the number of reads assigned to each OTU standardized with cumulative sum-scaling.

Fecal concentrations of EED biomarkers were used as continuous variables. These included calprotectin (in µg/g) as a measure of intestinal inflammation. Calprotectin is an unspecific marker that has been widely used to diagnose and monitor inflammatory bowel diseases in children and that has been associated with geophagy and stunting in the context of EED [8, 29–32]. Alpha-1-antitrypsin (in mg/dl), a serine protease inhibitor that is excreted in the gut during protein loss enteropathy, was used as a measure of intestinal permeability; and REG1B (in µg/g), a gene involved in the regeneration of intestinal cells, was used as a measure of intestinal damage [2, 33, 34].

### Statistical analysis

Variables that could confound the association between gut microbiota and EED biomarkers were included as covariates. These included season of fecal sample collection, exact age and sex of the child, delivery mode, maternal education, household-assets Z-score, ownership of domestic animals, source of drinking water, type of sanitary facility, randomization group and number of sequencing reads.

As primary analyses, we tested hypotheses on the association between gut microbiota diversity and maturity and markers of intestinal inflammation, permeability and damage. We also conducted exploratory, secondary analyses of other measures of microbial community composition. Because the sampling time points were 12 months apart and microbiota composition and levels of EED biomarkers change rapidly at this age, no longitudinal analyses were conducted. All analyses were cross-sectional (separate analyses for the 3 time points) and the main time point was 18 months. At this age, children are relatively mobile and no longer predominantly breast-fed, which increases potential exposure to pathogens in the living environment [29]. Analyses with 6 and 30 months data were additionally performed to examine whether the associations between microbiota and EED vary at different ages. At each time point, all available samples were analyzed and participants who missed a visit were included in the analyses at other time points.

The hypothesis that there is an inverse association between the diversity and maturity of fecal microbiota and concentrations of EED biomarkers was tested using linear regression models. Calprotectin, alpha-1-antitrypsin and REG1B were used as outcomes and MAZ-score and Shannon Index as predictors in fully covariate-adjusted models (forced-entry). Outcome variables were assessed for conformance to the normal distribution assumption by inspecting histograms. For calprotectin and alpha-1-antitrypsin, log(10)-transformed values were used in the analyses. Because breast-feeding has been found to be associated with both microbiota maturity and diversity and EED biomarkers, a sensitivity analysis was completed to assess interaction (at *p* < 0.1 for the interaction term) or confounding (at >10% change-in-estimate) by breast-feeding status at 18 months [35]. At 6 months, almost all children in this population are breast-fed and no data on breast-feeding were collected at 30 months. We also conducted a sensitivity analysis excluding children whose mothers were HIV-positive during pregnancy. HIV tests were not conducted on children.

Secondary analyses on beta diversity between participants with different levels of EED biomarkers assessed differences in weighted and unweighted UniFrac distances with permutational analysis of variance (PERMANOVA). The models included all covariates aforementioned and assessed the marginal effect of calprotectin, alpha-1-antitrypsin and REG1B [36]. Pseudo *p* values were obtained through 1000 permutations.

To analyze associations at the level of individual bacterial taxa, Random Forests machine learning regression models were built to estimate how relative abundances of bacterial OTUs at 18 months predict the levels of calprotectin, alpha-1-antitrypsin and REG1B. OTUs were ranked based on their importance, which was measured as the mean decrease in prediction accuracy if the OTU was excluded from the model. Only OTUs present in at least 10% of the samples were included in the models. For the 10 highest ranked OTUs in each of the 3 models, differences in EED-biomarker abundances between participants with relative taxon abundances below and above median were compared with Mann–Whitney test and fdr-corrected *p* values were calculated using the Benjamini–Hochberg correction.

All analyses were carried out in STATA version 15 and R version 3.5.3.

### Ethics approval

The iLiNS-DYAD study was approved by the College of Medicine Research and Ethics Committee, Malawi and the ethics committee of the Pirkanmaa hospital district, Finland. All participants provided informed consent at enrollment by signing or thumb printing a consent form.

## RESULTS

**Fig. 1.**
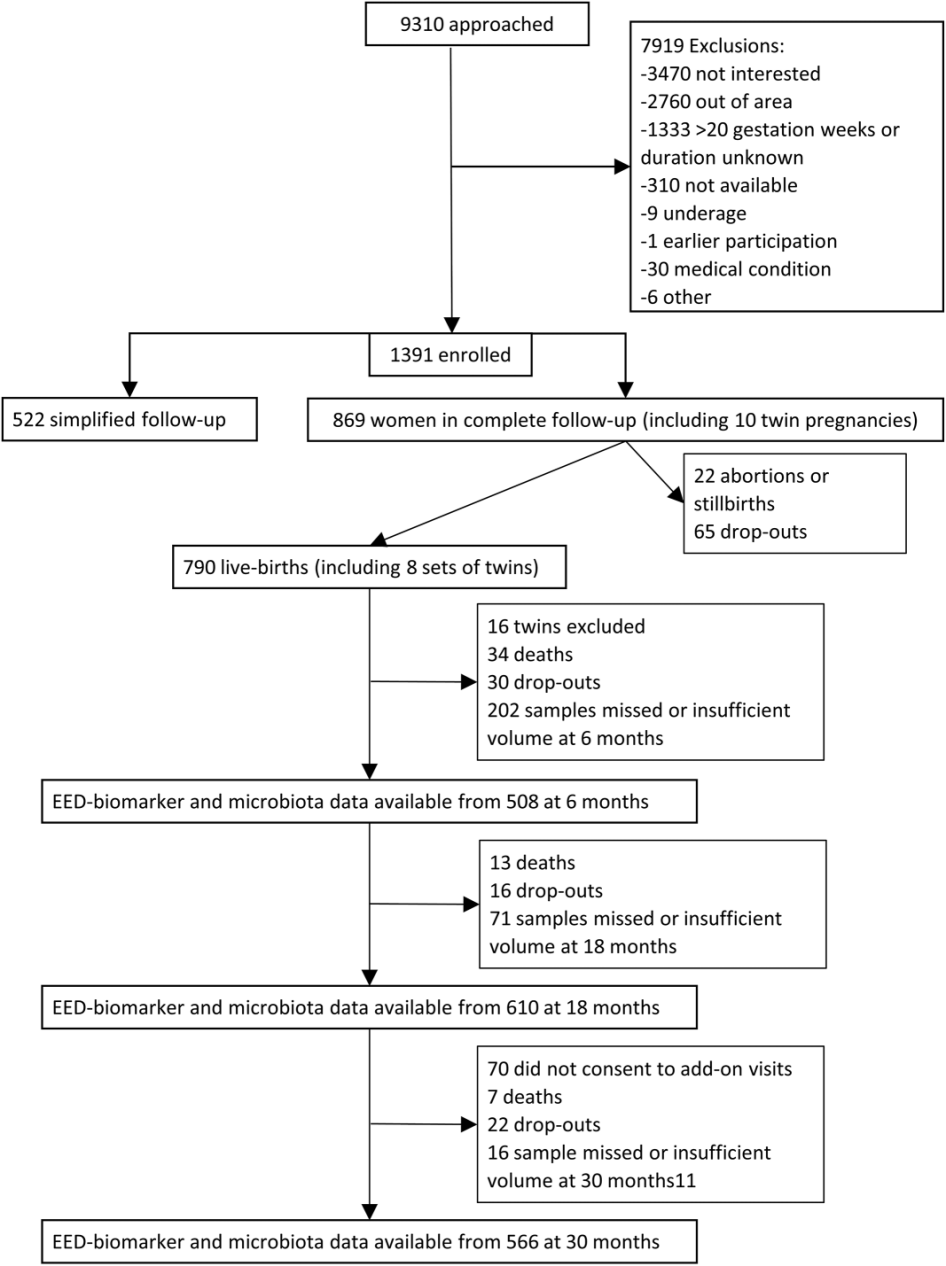
Participant flow.

**Fig. 2.**
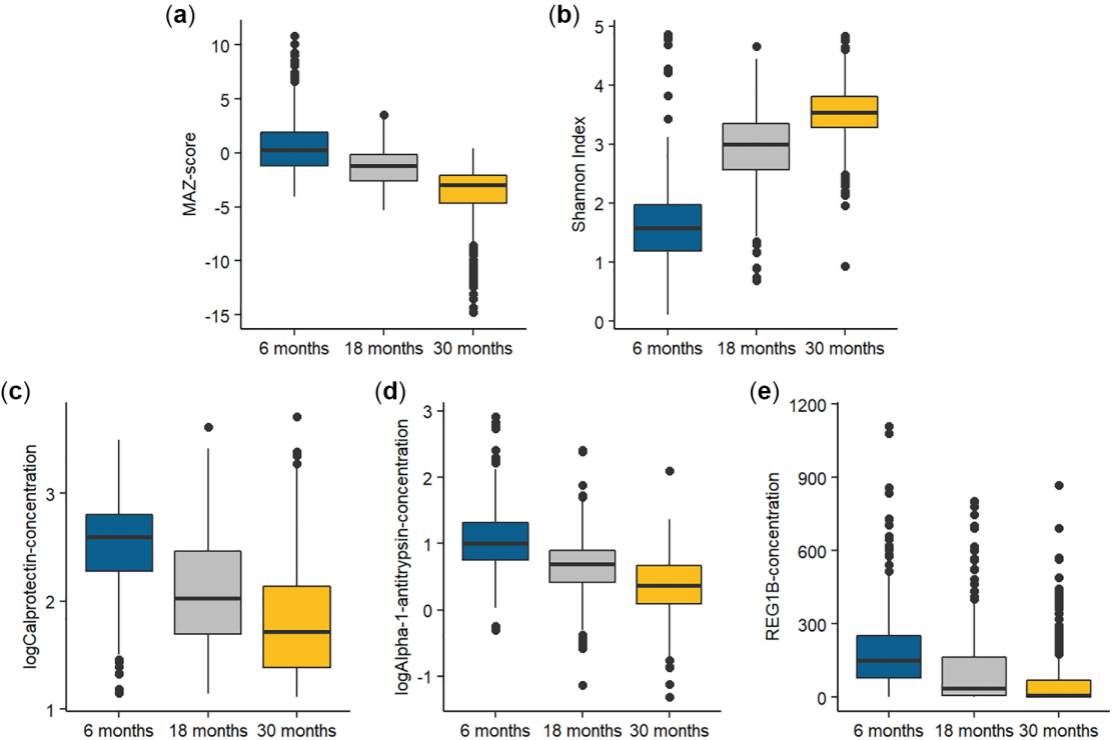
Distribution of microbiota maturity and diversity variables and EED biomarkers.

There were 790 live-born children (including 8 sets of twins, who were excluded from the analyses) in the complete follow-up scheme, of which 611, 666 and 596 provided a fecal sample at 6, 18 and 30 months, respectively. Of these, complete data for predictors and at least 1 EED biomarker were available for 459, 578 and 532 at 6, 18 and 30 months, respectively. A total of 77 children died between birth and 30 months, 68 were otherwise lost to follow-up and 71 did not consent to additional follow-up beyond 18 months (Fig. 1). Participants included in the analysis had a median gestational age of 39.7 weeks and a birth length of 49.8 cm. Thirteen percent had access to piped water and 10% had improved sanitary facilities. Excluded participants had lower maternal age, higher maternal education, lower gestational age and length at birth, and higher socioeconomic status compared to participants who provided microbiota and EED data (Table 1). The fecal concentrations of all 3 EED biomarkers decreased with age (Fig. 2, Supplementary Table S1).

**Table 1:** Characteristics of included and excluded participants, median (inter-quartile range) or percentage

Characteristic	Included	Excluded	
Participants, *n*	610	180	*p* value
Maternal age at enrollment, years	24.9 (20.4; 29.5)	23.0 (19.3; 28.1)	0.02
Maternal education completed, years	3 (0; 6)	4 (1; 7)	0.03
Positive malaria RDT of the mother at enrollment	23%	23%	1.0
Mother HIV-positive at enrollment	12%	12%	0.82
Gestational age at birth, weeks	39.7 (38.7; 40.7)	39.2 (37.6; 40.3)	<0.001
Length at birth, cm	49.8 (48.5; 51.3)	49.0 (47.6; 50.3)	<0.001
LAZ at birth	−0.9 (−1.6; −0.3)	−1.4 (−2.2; −0.7)	<0.001
Household assets Z-score	−0.4 (−0.7; 0.1)	0.2 (−0.7; 0.8)	0.001
Source of drinking water is borehole, well, river, or lake (vs. piped)	87%	75%	<0.001
Type of sanitary facility is none or regular pit latrine (vs. ventilation improved pit latrine or water closet)	90%	91%	0.62

LAZ: length-for-age Z-score; RDT: rapid diagnostic test.

Included participants are those who had data on calprotectin and microbiota maturity and diversity available at 18 months. *p* values are obtained from Mann–Whitney test (continuous variables) or chi-square test (proportions).

**Fig. 3.**
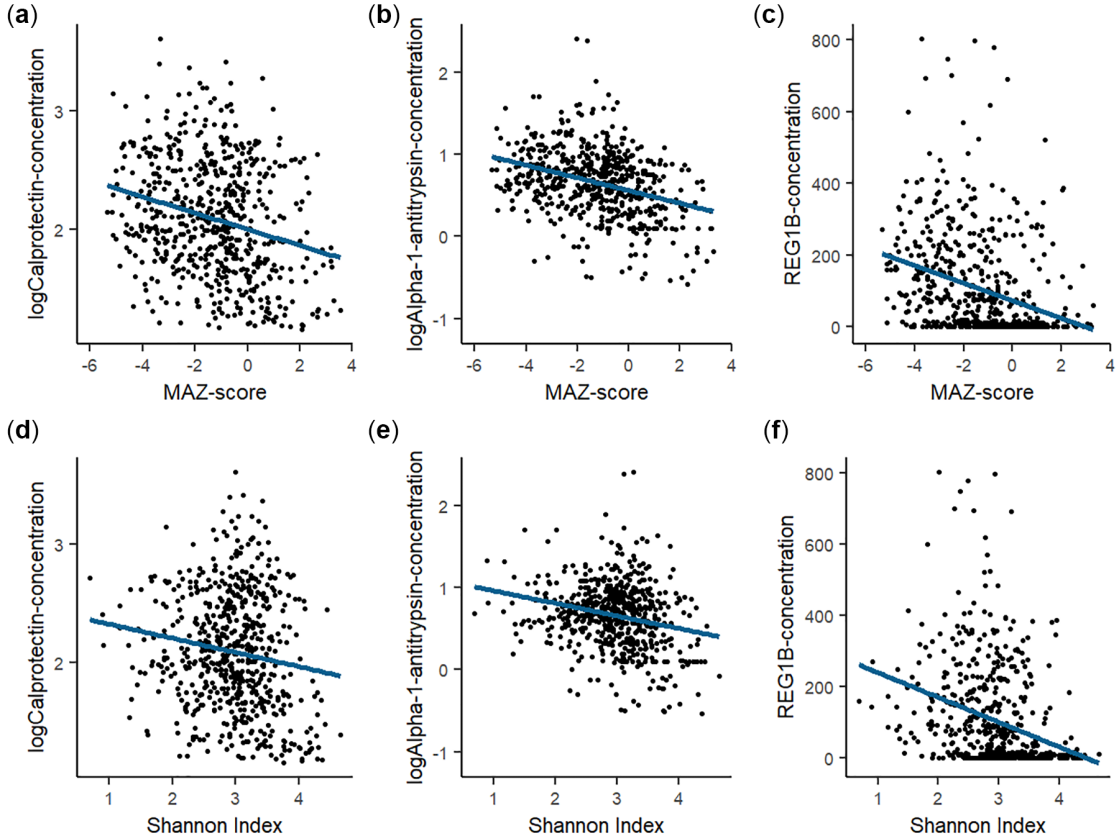
The association of microbiota maturity and diversity variables with fecal calprotectin, alpha-1-antitrypsin and REG1B at 18 months. Scatterplots of MAZ-score (microbiota-for-age Z-score) (A–C) or Shannon Index (D–F) and EED biomarkers at 18 months with fitted (unadjusted) linear regression lines.

Log-transformed concentrations of calprotectin and alpha-1-antitrypsin and the concentration of REG1B were inversely associated with MAZ-score and Shannon Index at 18 months (each *p* ≤  0.001; Table 2 and Fig. 3). The results were similar at 30 months (Supplementary Table S2). At 6 months, calprotectin was inversely associated with MAZ-score, alpha-1-antitrypsin was inversely associated with both MAZ-score and Shannon Index and REG1B was not associated with either (Supplementary Table S3). At 18 months, 8% of the children were no longer breast-fed. When breast-feeding status was added as an additional covariate to the models at 18 months, the point estimates changed by less than 10% and the interaction term for breast-feeding status was not statistically significant in any of the models (*p* > 0.1). An analysis that excluded children of HIV-positive mothers gave essentially identical regression coefficients to those in the main analysis for all outcomes (data not shown).

**Table 2:** The association of microbiota maturity and diversity variables with fecal calprotectin, alpha-1-antitrypsin and REG1B at 18 months.

	Association between predictor and outcome variable, adjusted for covariates^a^								
	Log(calprotectin)			Log(alpha-1-antitrypsin)			REG1B		
Predictor variable	Regression coefficient (95% CI)	*p* value	*n*	Regression coefficient (95% CI)	*p* value	*n*	Regression coefficient (95% CI)	*p* value	*n*
MAZ-score	−0.07	<0.001	578	−0.08	<0.001	547	−25.14	<0.001	558
	(−0.09, −0.05)			(−0.1, −0.06)			(−31.75, −18.52)		
Shannon Index	−0.12	<0.001	578	−0.16	<0.001	547	−71.96	<0.001	558
	(−0.18, −0.05)			(−0.22, −0.11)			(−91.22, −52.70)		

Results from multivariable analysis. CI: confidence interval; MAZ: microbiota-for-age Z-score.

a Adjusted for education level of the mother, household assets index, water source, sanitary facility, domestic animals, season, sex of the child, delivery mode, exact age, randomization group, sample processing pool and sequencing depth.

**Fig. 4.**
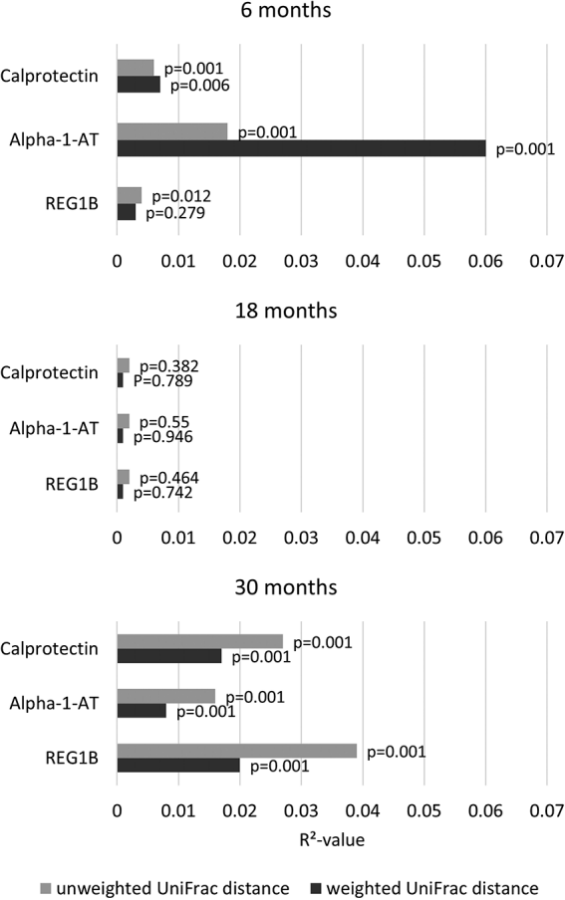
Associations between EED biomarkers and phylogenetic dissimilarity. Bar chart of *R*^2^ values from covariate-adjusted PERMANOVA models. Pseudo *p* values were obtained through 1000 permutations.

In secondary analyses on beta diversity, phylogenetic distance measured as UniFrac distance was not associated with any EED biomarker at 18 months (pseudo *p* values > 0.05). At 30 months both unweighted and weighted UniFrac distances were associated with all EED biomarkers with *R*^2^ values ranging from 0.8% to 3.9%. At 6 months unweighted UniFrac distances were associated with concentrations of all EED biomarkers and weighted UniFrac distances were associated with calprotectin and alpha-1-antitrypsin concentration with *R*^2^ values between 0.4% and 6% (Fig. 4).

**Fig. 5.**
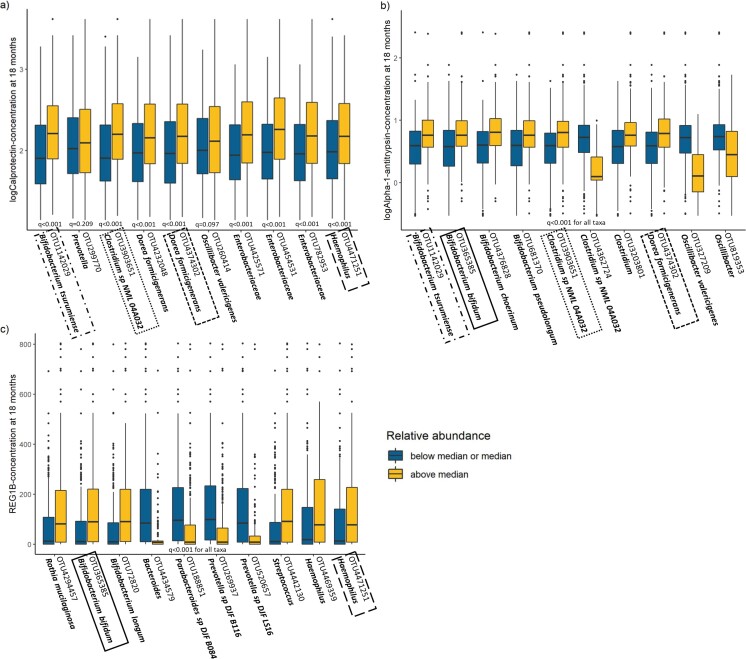
Differences in EED biomarker concentrations by relative taxon abundance at 18 months. Box plots of (log-transformed) concentrations of calprotectin, alpha-1-antitrypsin and REG1B at 18 months over relative abundances of bacterial taxa (taxon abundance below or at median vs. above median). Taxa that were among the 10 most important OTUs in predicting EED biomarker concentrations at 18 months in Random Forests models are listed. (A) shows taxa that were ranked highest in predicting calprotectin concentration, (B) shows taxa that were ranked highest in predicting alpha-1-antitrypsin concentration and (C) shows taxa that were ranked highest in predicting REG1B concentration. Taxa in frames were among the highest ranked taxa in 2 of the 3 models. *q* values are fdr-corrected *p* values from Mann–Whitney test.

Random Forests models on individual bacterial taxa (OTUs) predicting the concentration of EED biomarkers at 18 months explained 24.6%, 27.7% and 24.5% of the variation in the concentrations of calprotectin, alpha-1-antitrypsin and REG1B, respectively. Taxa that were among the 10 highest ranked predictors in at least 1 of the models included OTUs assigned to the bacterial orders of Actinomycetales, Bifidobacteriales, Bacteroidales, Clostridiales, Lactobacillales, Enterobacteriales and Pasteurellales. Out of all Bifidobacteriales taxa present at 18 months, 42% (5/12) were among the highest ranked predictors of EED biomarker concentrations. Differences in EED biomarker concentrations by relative taxon abundance are shown in Fig. 5.

## DISCUSSION

The aim of this study was to investigate the associations between biomarkers of EED and gut microbiota composition in rural Malawian children. Our findings support the hypothesis of an inverse association between the biomarkers calprotectin, alpha-1 antitrypsin and REG1B, and microbiota maturity and diversity at 18 months of age. The findings were similar in children aged 6 and 30 months. In secondary analyses, levels of EED biomarkers were associated with microbial beta-diversity measured as UniFrac distances at 6 and 30, but not at 18 months. In addition, we identified bacterial taxa that predicted the levels of EED biomarkers, including several Bifidobacterium, Enterobacteriacea, Dorea formicigenerans and Haemophilus taxa positively correlated and Prevotella and Oscillibacter taxa inversely correlated with EED biomarkers.

Our study is limited by the use of microbiota data from fecal samples as a proxy for gut microbiota composition, because EED is a condition of the small intestine and the microbiota composition changes along the gastrointestinal tract [37]. In our study population of relatively healthy children, it would not have been feasible nor ethical to perform endoscopies to obtain samples from the small intestine [38]. Even though previous studies with sampling from the small intestine found the differences between small intestinal and fecal microbiota, in those studies many bacterial taxa from the small intestine were also detected in feces and disease-associated changes occurred in both small intestinal and fecal microbiota [15, 37]. Thus, the observed differences in microbiota composition likely reflect EED-associated intestinal dysbiosis, but we cannot infer whether these differences are causally related to EED.

Further, we did not collect detailed information on nutrient or breast-milk intake at all time points [39, 40]. However, sensitivity analyses did not suggest confounding or interaction by breast-feeding status at 18 months and previous studies in this population have revealed relatively homogenous breast-feeding and complementary feeding practices [41, 42]. Therefore, we do not expect our conclusions to be significantly biased by differences in feeding patterns. Participants who were lost to follow-up and did not provide data for these analyses had on average lower gestational age and length at birth and a higher socioeconomic status than included study participants. Theoretically, this may reduce the external validity of our results. However, although the differences were statistically significant, they were very small and clinically probably insignificant. Therefore, we believe that the included study participants are representative of the study population. Finally, we used only calprotectin as a marker of intestinal inflammation and did not analyze other established markers such as myeloperoxidase, which might have limited our ability to detect the differences in microbiota composition related to intestinal inflammation [43, 44].

The presented findings are mostly consistent with reports from the previous studies. A recent study found an inverse association between microbiota diversity and fecal neopterin concentration [45]. Otherwise, the association between microbiota diversity and maturity and EED biomarkers has not been described before, but reduced microbiota diversity and maturity have been associated with malnutrition and reduced diversity has been associated with disease severity in patients with Crohn’s disease [13, 20, 46, 47]. One smaller study on EED conducted in Malawi measured the lactulose to mannitol ratio in young children and did not find associations with microbiota diversity. Further, the study reported the differences in relative abundances of bacterial taxa that where not found to predict EED biomarkers in our study [17]. This discrepancy could be due to different sample processing and analysis methods, but it is also possible that the biomarkers used in our study capture different aspects of EED than the lactulose to mannitol ratio.

Regarding differences in bacterial taxon abundances, the finding of a positive association between several Bifidobacterium taxa and all 3 EED biomarkers is unexpected, because Bifidobacterium species have previously been associated with probiotic and anti-inflammatory properties [20, 48, 49]. However, the relative abundance of Bifidobacteria also decreases significantly with age and a higher abundance can thus reflect an EED-associated immaturity of the microbiota [24, 50]. This has previously been inversely associated with ponderal growth and might be attributed to a reduced fitness of certain Bifidobacteria strains [51, 52]. Our finding of a positive association between Enterobacteriaceae and calprotectin is in line with several studies linking Enterobacteriaceae with intestinal inflammation as well as HIV, which often leads to a form of enteropathy [20, 53–56]. We found both positive and negative associations between Clostridium taxa and EED biomarkers, in agreement with several studies reporting opposing functions of different Clostridium species [57]. Haemophilus, Streptococcus and Rothia mucilaginosa, which were positively associated with calprotectin and REG1B, are part of the oral microbiota [58, 59]. Decompartmentalization of the microbiota, that is oral taxa present in the distal gastrointestinal tract, has been associated with inflammation and stunting [15, 37, 53].

There are several plausible mechanisms by which the gut microbiota could cause or influence EED or by which EED could lead to changes in microbiota composition. Pathogenic bacteria could directly cause EED through mucosal damage or enteroinvasion and intestinal inflammation [60–62]. Though we could not directly assess the associations between pathogenic bacteria and EED biomarkers due to insufficient taxonomic resolution at species level, several EED-associated taxa were assigned to the family of Enterobacteriaceae, which includes pathogenic *Escherichiacoli* and Shigella species [63]. Further, members of the intestinal microbiota could indirectly alleviate or aggravate EED through interactions with the immune system. Potential mechanisms include production of anti-inflammatory short-chain fatty acids, strengthening of the epithelial barrier, and induction of immune cells [53, 64–67]. On the other hand, antimicrobial peptides and immunoglobulin A secreted by epithelial cells after sensing of bacteria can affect the microbiota composition [16, 68]. Of the EED biomarkers used in this study, calprotectin and alpha-1 antitrypsin have known antibacterial properties and REG1B is thought to be antibacterial based on similarities with other antimicrobial peptides, implying that these biomarkers could directly affect microbiota composition [34, 69–73]. Finally, changes in bile-acid metabolism have been described in EED and these changes could mediate influences of the gut microbiota on host metabolism [74, 75]. Thus, intestinal dysbiosis could be either a cause or a consequence of EED. It is also conceivable that EED-associated changes in microbiota composition constitute beneficial adaptations that mitigate the negative effects of the disease [76, 77].

Our findings support the hypothesis of an association between the gut microbiota composition and EED assessed by the biomarkers calprotectin, alpha-1 antitrypsin and REG1B in rural Malawian children. EED was found to be associated with reduced microbiota diversity and maturity and relative abundances of potentially pathogenic and oral bacterial taxa. However, the etiology of EED may vary in different settings and microbiota composition has been shown to differ by geographic location [78]. Therefore, the findings of our study are likely not generalizable to other regions. To advance the prevention and treatment of EED, further studies are needed to establish whether these associations are causal and whether similar patterns occur in different populations.

## Supplementary Material

fmac012_Supplementary_Data
