# Correction to: Associations between Gut Microbiota and Intestinal Inflammation, Permeability and Damage in Young Malawian Children

**DOI:** 10.1093/tropej/fmac071

**Published:** 2022-08-13

This is a correction to: Emma Kortekangas, MD, Yue-Mei Fan, PhD, David Chaima, PhD, Kirsi-Maarit Lehto, PhD, Chikondi Malamba-Banda, MPhil, Andrew Matchado, PhD, Chilungamo Chingwanda, PhD, Zhifei Liu, MPH, Ulla Ashorn, PhD, Yin Bun Cheung, PhD, Kathryn G Dewey, PhD, Kenneth Maleta, PhD, Per Ashorn, PhD, Associations between Gut Microbiota and Intestinal Inflammation, Permeability and Damage in Young Malawian Children, *Journal of Tropical Pediatrics*, Volume 68, Issue 2, April 2022, https://doi.org/10.1093/tropej/fmac012

In the originally published version of this manuscript, the boxes in subfigure (b) of Figure 5 were in the wrong order.

This error has been corrected.

