# Supplementary material for: Associations between Gut Microbiota and Intestinal Inflammation, Permeability and Damage in Young Malawian Children
Source: J Trop Pediatr. 2022 Feb 12;68(2):fmac012. doi: 10.1093/tropej/fmac012 (PMC8846364; doi:10.1093/tropej/fmac012)
Supplement: fmac012_Supplementary_Data [file fmac012_supplementary_data.zip › Kortekangas Gut microbiota and EED_Supplementary Table 3.docx]

Supplementary Table 3. The association of microbiota maturity and diversity variables with fecal calprotectin, alpha-1-antitrypsin, and REG1B at 6 months. Results from multivariable analysis.

|  | Association between predictor and outcome variable, adjusted for covariates^a^ | | | | | | | | |
| --- | --- | --- | --- | --- | --- | --- | --- | --- | --- |
| Predictor variable | Log(Calprotectin) | | | Log(Alpha-1-antitrypsin) | | | REG1B | | |
|  | Regression coefficient (95% CI^b^) | P-value | n | Regression coefficient (95% CI) | P-value | n | Regression coefficient (95% CI) | P-value | n |
| MAZ-Score^c^ | -0.03  (-0.05,-0.02) | <0.001 | 459 | -0.04  (-0.05,-0.02) | <0.001 | 406 | -0.22  (-6.18,5.74) | 0.942 | 426 |
| Shannon Index | -0.06  (-0.13,0.00) | 0.067 | 459 | -0.14  (-0.22,-0.05) | 0.001 | 406 | 11.37  (-15.97,38.70) | 0.414 | 426 |

^a^ adjusted for education level of the mother, household assets index, water source, sanitary facility, domestic animals, season, sex of the child, delivery mode, exact age, randomization group, sample processing pool, sequencing depth

^b^ confidence interval

^c^ microbiota-for-age Z-score
