# Supplementary material for: Associations between Gut Microbiota and Intestinal Inflammation, Permeability and Damage in Young Malawian Children
Source: J Trop Pediatr. 2022 Feb 12;68(2):fmac012. doi: 10.1093/tropej/fmac012 (PMC8846364; doi:10.1093/tropej/fmac012)
Supplement: fmac012_Supplementary_Data [file fmac012_supplementary_data.zip › Kortekangas Gut microbiota and EED_Supplementary Table 1.docx]

Supplementary Table 1. Concentrations of fecal EED biomarkers at 6, 18, and 30 months, median (inter-quartile range)

|  | 6 months | 18 months | 30 months |
| --- | --- | --- | --- |
| Calprotectin | 395 (189;643) µg/g | 107 (5;293) µg/g | 52 (24;139) µg/g |
| alpha-1-antitrypsin | 10 (6;21) mg/dl | 5 (3;8) mg/dl | 2 (1;5) mg/dl |
| REG1B | 152 (80;255) µg/g | 38 (7;164) µg/g | 8 (0;71) µg/g |
